# Social health gradient and risk factors among patients hospitalized for COVID-19 and pre-pandemic respiratory infections. A linked national individual case-control study in Belgium

**DOI:** 10.3389/fpubh.2024.1426898

**Published:** 2024-10-28

**Authors:** Arnaud Bruyneel, Jérôme E. Dauvergne, Nicolas Dauby, Jean-Christophe Goffard, Andrea Rea, Judith Racape

**Affiliations:** ^1^Hospital Management and Nursing Research Department, Research Center in Health Economics, School of Public Health, Université Libre de Bruxelles (ULB), Brussels, Belgium; ^2^Department of Anaesthesiology and Critical Care, CHU Nantes, Laënnec Hospital, Nantes Université, Nantes, France; ^3^Department of Infectious Diseases, Centre Hospitalier Universitaire (CHU) Saint-Pierre, Université Libre de Bruxelles (ULB), Brussels, Belgium; ^4^Research Center in Environmental Health, School of Public Health, Université Libre de Bruxelles (ULB), Brussels, Belgium; ^5^Department of Internal Medicine, Erasmus Hospital, Université Libre de Bruxelles, Brussels, Belgium; ^6^Group for Research on Ethnic Relations, Migrations and Equality, Faculty of Philosophy and Social Sciences, Université Libre de Bruxelles (ULB), Brussels, Belgium; ^7^Chair in Health and Precarity, Faculty of Medicine, Université Libre de Bruxelles (ULB), Brussels, Belgium; ^8^Research Center in Epidemiology, Biostatistics and Clinical Research, School of Public Health, Université Libre de Bruxelles (ULB), Brussels, Belgium

**Keywords:** COVID-19, health status disparities, risk factors, socioeconomic factors, hospitalization, length of stay

## Abstract

**Introduction:**

The literature establishes a clear social gradient in health for transmissible respiratory diseases. However, this gradient’s extent remains largely unexplored in the context of COVID-19, and it is uncertain whether the pandemic has exacerbated this gradient. The study aims to compare the socio-economic profiles and comorbidities during the COVID-19 pandemic with a control population affected by viral pneumonia/respiratory disease in 2019.

**Methods:**

This case-control study analyzed linked data from all patients hospitalized for COVID-19 in 2020 (*n* = 22,087) and for respiratory diseases in 2019 (*n* = 7,586). Socio-economic data from the social security database were linked to clinical data from the hospital registry. We analyzed the socio-demographic and clinical factors associated with COVID-19 hospitalization (control group, wave 1, and wave 2) using multinomial regressions and logistic regression models and the length of stay during hospitalization using binomial negative regressions.

**Results:**

A social health gradient was observed in both the COVID-19 and control groups, with a significant increase across waves for COVID-19 (p-trend < 0.0001). Men, people over the age of 45, those with comorbidities, high population density, lower income, lower socio-economic status, and people living in Brussels capital were at higher risk of COVID-19 hospitalization and longer length of stay compared to the control group. Except for sub-Saharan Africans, all patients of foreign nationality had a significantly increased risk of hospitalization (*p* < 0.001), but a shorter length of stay compared to Belgians.

**Conclusion:**

The socio-health gradient for COVID-19 followed the same pattern as that observed in pre-pandemic respiratory diseases, intensifying in the second wave and among the most deprived groups. This study emphasizes the importance of collecting social data alongside clinical data for a better understanding of social health inequalities and for tailoring health prevention policies.

## Highlights

- The social health gradient observed in COVID-19 mirrors that of pre-pandemic respiratory diseases.
- Social health gradient exacerbation was more pronounced in the most deprived groups and increased between the first 2 waves of COVID-19.
- Risk factors are not always consistent between risk of hospitalization and increased length of stay.

## Introduction

Scientific literature has repeatedly demonstrated that socio-economic factors are crucial determinants of health-related outcomes. Socio-economic profiles may be considered as one of the main causes of health disparities between different population groups (1–3). For example, in several European countries, mortality rates are twice as high for individuals in the lowest educational groups compared to the highest. Across European countries, individuals with lower education levels present lower self-rated health, compared to those with higher levels of education (4, 5).

Before COVID-19, studies had identified an association between socio-economic profiles and several healthcare outcomes, including increased frequency of hospital admissions and intensive care unit (ICU) admissions, higher rates of avoidable hospitalizations, a longer length of stay, and a higher hospital mortality rate (6–9). These adverse outcomes had several explanations, as the reasons were many. The underlying mechanisms contributing to this heightened risk included varying exposures to transmissible respiratory diseases, heightened susceptibility to infectious diseases and associated complications, health risk factors (such as tobacco and alcohol use), lower health literacy, lower vaccine coverage/ acceptability, and an unequal access to healthcare (3, 10). Some argued that the COVID-19 pandemic was experienced as a “syndemic pandemic” (11, 12). Social epidemiology indeed suggests that infections and deaths from COVID-19 occur along existing axes of social inequalities, and individuals from ethnic minority backgrounds, disadvantaged socio-economic backgrounds and deprived areas are more likely to be affected (13, 14). The relationship between inequalities and communicable respiratory diseases (such as influenza, tuberculosis, etc.) (15), as well as the social determinants of health linked to chronic diseases, have been widely debated in the literature (16). During the pandemic’s various waves, the interaction and exacerbation of existing socio-economic inequalities, case rates, symptom severity, and morbidity and mortality of infectious diseases remained largely under-explored, especially for the most vulnerable populations (17). In reality, it is still unclear whether the well-established social health gradient among respiratory diseases was also present for COVID-19, or whether it was exacerbated. Pre-pandemic control groups, useful to demonstrate the association between patient outcomes and socio-economic profiles during the COVID-19 pandemic, are most often lacking (18–20).

Belgium experienced one of the most serious epidemics of COVID-19 in Europe during the first two waves, with a high monthly excess mortality rate (21, 22). In Belgium, the influence of household composition and age profiles on the income gradient was observed in COVID-19-related deaths (23). However, this profile resembled the all-cause mortality gradient in the non-pandemic period (23). The most disadvantaged municipalities and migrants were likely to experience a higher incidence of COVID-19 (24–26), but this socio-economic pattern was specifically linked to COVID-19. A lack of social data in clinical databases means that we do not have access to the overall clinical and social profiles of hospital patients belonging to this population, which often remains “invisible” to the country as a whole.

The peak of the first wave of the pandemic occurred around the 10th of April, 2020, with over 1,500 patients hospitalized in ICUs, and capacity increased to 2,000 ICU beds (27). The second wave, stronger and more prolonged, peaked at the beginning of November 2021 (28). Unlike the first wave, hospital activity continued during the second wave, placing additional strain on hospital capacity and healthcare staff (29). A study noted significant changes in COVID-19 hospitalizations’ clinical characteristics between epidemic waves, but did not analyse socio-demographic factors (30). Furthermore, studies conducted in Brussels only highlighted a strong association between socio-economic profiles and hospitalizations for COVID-19 in patients under 65 (legal retirement age in Belgium), and who presented different risk factors (19, 31).

Due to the lack of comprehensive individual-level social and clinical data, the overall clinical and social profile of patients hospitalized with COVID-19 has not been adequately analyzed. Additionally, studies of socio-economic profiles and their outcomes often lack a control group with other well-known respiratory diseases, which is crucial for understanding the social gradient in health. To address these gaps, we conducted this study.

The aims of this study were to compare socio-economic profiles and co-morbidities during the COVID-19 pandemic with a control population affected by viral pneumonia/respiratory diseases in 2019, and to explore their association with outcomes for individuals aged under 65 years old, including hospitalization and length of stay.

## Materials and methods

### Research hypotheses

Two hypotheses will be tested in this study. The first hypothesis is that the well-known socio health gradient during the COVID-19 pandemic followed a similar pattern to that observed for respiratory diseases in the pre-pandemic period. The second hypothesis is that the risk factors for hospitalization and length of stay were similar between two populations, COVID-19 and respiratory diseases.

### Study population

Data covered all hospitalized COVID-19 patients between the 1st of January 2020 and the 31st of December 2020 (*n* = 22,087), and all patients hospitalized for viral pneumonia and respiratory diseases between the 1st of January 2019 and the 31st of December 2019 (*n* = 7,586) in Belgium. The group of patients hospitalized for COVID-19 in 2020 is named “COVID-19 population,” while the group of patients hospitalized for viral pneumonia and respiratory diseases in 2019 is named “control group.” Data is provided after exclusion of under-18 s and pregnant women. Data is provided after the exclusion of individuals under 18 and pregnant women. Pregnant women were excluded because the database did not allow differentiation between hospitalizations due to childbirth and those resulting from COVID-19. Children were excluded due to the low number of COVID-19 hospitalizations and the lack of socio-economic data linked to their parents, which made it impossible to conduct analyses for this population. We excluded those above 65 years (*n* = 61,109) and patients hospitalized in both groups (*n* = 1,101). We therefore had a control group (*n* = 7,586) and a COVID-19 group (*n* = 22,087) (Supplementary Figure S1).

We divided the 2020 COVID-19 population into two waves: wave 1 from the 1st of January 2020 to the 30th of June 2020 (*n* = 9,668), and wave 2 from the 1st of July 2020 to the 31st of December 2020 (*n* = 12,419).

### Data collection

Data came from the Minimal Clinical Data (MCD), a registration system through which all non-psychiatric hospitals in Belgium must make their (anonymised) administrative, medical and nursing data available to the Federal Public Service (FPS)’s Public Health department. The MCD covers all admissions in Belgian hospitals and contains patient data, admission data and an unlimited number of diagnoses and procedures. Each hospital admission gives rise to a record (MCD). Diagnoses made during hospitalization are coded according to the ICD-10-CM classification (International Classification of Diseases, 10th Revision, Clinical Modification). Diagnoses are categorized into “main diagnosis”—defined as the pathology explaining patient hospitalization (linked to the admission diagnosis)—and “secondary diagnoses” such as other diagnoses, for example co-morbidities (32, 33).

During the COVID-19 pandemic, a special code was introduced by the National Institute for Health and Disability Insurance (NIHDI) to identify COVID-19. We analyzed admissions using codes B97.29 (ICD-10-CM) plus the NIDHI code. For the control group (2019), we selected admissions for viral pneumonia and respiratory infections (codes ICD-10-CM: J12/J20-22/J18/J80/J96-98/B34/B97).

The Crossroads Bank of Social Security (CBSS) electronically gathers socio-economic data originating from social security administrations in Belgium. Each organization is in charge of recording and updating their own data (34).

The linkage was carried out by the trusted third-party, the national digital health data platform. Each requested variable was discussed and approved by the data managers of the CBSS and MCD. Small cell risk analyses were conducted to minimize the risk of identifying individuals. Obtaining linked databases is a process that is regulated by the Belgian Information Security Committee. Each individual residing in Belgium is identified with a unique pseudonymised number (national register number). This number allows to link the two databases used for the study.

### Variables

Four types of data were extracted to analyse socio-economic profiles. The socio-economic status (SES) is a statistical indicator combining housing, education level and income. We categorized this indicator in 5 groups from the most deprived (group 1) to the least deprived (group 5). Population density was provided by BCSS as categorized in 3 groups according to population density in 2019: 1/ low (under 8,800) 2/ medium (8,800 to 14,300) 3/ high (over 20,400 inhabitants/km^2^). Number of people per household is categorized in 4 groups: 1, 2, 3–4 and more than 4 people per household. Income was provided in deciles and was divided into quintile: from highest (Quintile 1) to lowest (Quintile 5).

The socio-demographic data was extracted from the CBSS and includes: age, sex, first nationality, and region. Age was categorized into two groups: under 45 and over 45 years old. We used the first nationality and classified into six groups as the most represented in Belgium: Belgian, EU 28, North Africa, Sub-Saharan Africa, Middle East and other. Belgium is divided into three regions: Wallonia, Flanders and Brussels-Capital.

For the main co-morbidities, ICD-10 codes were extracted from the MCD secondary diagnoses: diabetes (E08–E13; Diabetes mellitus), hypertension (I10–I16: Hypertensive diseases), obesity (E66: Overweight and obesity), renal disease (N17–N18: Acute kidney failure and chronic kidney disease), neoplasia (C00–C96: Malignant neoplasms), cardiovascular disease (I20–I25: Ischemic heart diseases, I60–I69: Cerebrovascular diseases, I70–I79: Diseases of arteries, arterioles and capillaries) and pulmonary disease (I26–I28: Pulmonary heart disease and diseases of pulmonary circulation, J40–J47: Chronic lower respiratory diseases).

The two outcomes studied—hospital admission and total length of stay in days—were exported from the MCD.

### Statistical analysis

Qualitative variables were presented with proportions and compared using the Pearson Chi^2^ test. Quantitative variables were presented with medians (P25–P75) and compared using the Kruskal-Wallis test. We presented the characteristics of the population by waves. We analyzed socio-demographic and clinical factors associated with 1/ COVID-19 hospitalization (control group, wave 1 and wave 2) using multinomial regressions, and 2/ length of stay during hospitalization using binomial negative regressions. Crude and adjusted Odds Ratios (OR) with a 95% Confidence Interval were derived from the logistic regressions. Crude and adjusted Relative Rate Ratios (RRR) with a 95% Confidence Interval were derived from the multinomial regressions. Crude and adjusted Incidence Rate Ratios (IRR) with a 95% Confidence Interval were derived from the binomial negative regressions. The Wald Chi^2^ test’s *p*-value was presented. Due to overdispersion, we used negative binomial regression for modeling count data of length of stay. Multivariate models were conducted by adjusting for all variables included in the univariate analysis. The adequacy of the model was checked using the Hosmer-Lemeshow test, and the variance inflation factor was assessed to avoid collinearity. The significance level was set at *α* = 0.05. Analyses were performed using the Stata software, version 17 and R software version 4.1.2 (R Core Team).

### Ethical considerations

The process of linkage was approved by the Information Security Committee (Ref CSI/CSSS/22/250). Following the practical small cell risk analyses, the national digital health data platform provided the data to the researcher.

## Results

### Population characteristics

The characteristics of patients hospitalized for COVID-19 (waves 1 and 2) and for viral pneumonia/respiratory illness in 2019 are depicted in Table 1. Men and the age category (≥45 years) are represented in a higher proportion during both COVID-19 waves, compared to women and people under 45 years old, respectively. The proportion of first nationalities differed between COVID-19 and the control group, with the proportion of hospitalized non-Belgians being higher in COVID-19 and more pronounced in the second wave (26.1% for the control group, 36.6% for the first wave and 43.9% for the second wave). For other nationalities, for example North Africans, the proportion in the control group rose from 5.6 to 12.6% in the second wave (*p*-trend *p* < 0.001).

**Table 1:** Characteristics of the control population and the population hospitalized for COVID-19 (wave 1 and 2).

	Control population (*n* = 7,586)	Wave 1 (*n* = 9,668)	Wave 2 (*n* = 12,419)	*p*-value
Sociodemographic data				
**Sex, *n* (%)**				
Women	3,589 (47.3)	4,094 (42.3)	4,981 (40.1)	<0.0001
Men	3,997 (52.7)	5,574 (57.7)	7,438 (59.9)	
**Age category, *n* (%)**				
<45 years	2,778 (36.6)	2,497 (25.8)	23,385 (27.3)	<0.0001
≥45 years	4,808 (63.4)	7,171 (74.2)	9,834 (72.7)	
**Region, *n* (%)**				
Brussels	532 (7.9)	1,640 (17.3)	2,723 (22.6)	<0.0001
Flanders	4,173 (61.6)	4,626 (48.9)	4,863 (40.3)	
Wallonia	2,073 (30.6)	3,197 (33.8)	4,468 (37.1)	
**First nationalities, *n* (%)**				
Belgian	5,018 (73.9)	6,108 (64.4)	6,784 (56.1)	<0.0001
EU28	724 (10.7)	1,189 (12.5)	1,715 (14.2)	
North Africa	378 (5.6)	690 (7.3)	1,524 (12.6)	
Sub Saharan Africa	151 (2.2)	687 (7.2)	544 (4.5)	
Middle east	298 (4.4)	443 (4.7)	908 (7.5)	
Other	223 (3.3)	362 (3.8)	609 (5.0)	
Co-morbidities				
**Obesity, *n* (%)**	954 (12.6)	1,377 (14.2)	2,147 (17.3)	<0.0001
**Hypertension, *n* (%)**	1,387 (18.3)	2,497 (25.8)	3,251 (26.2)	<0.0001
**Diabetes, *n* (%)**	846 (11.1)	1,404 (14.5)	2,220 (17.9)	<0.0001
**Renal disease, *n* (%)**	537 (7.1)	819 (8.5)	920 (7.4)	0.001
**Neoplasm, *n* (%)**	1,314 (17.3)	897 (9.3)	1,822 (8.2)	<0.0001
**Cardio-vascular disease, *n* (%)**	1,282 (16.9)	1,823 (18.9)	2,007 (16.2)	<0.0001
**Pulmonary diseases, *n* (%)**	1,441 (19.0)	1,546 (16.0)	1,872 (15.1)	<0.0001
**Smoking, *n* (%)**	1,789 (23.6)	1,064 (11.0)	1,136 (9.1)	<0.0001
Clinical outcomes				
ICU admission, *n* (%)	616 (8.1)	1,975 (20.4)	2,214 (17.8)	<0.0001
**Length of stay (days)**				
Median (P25–P75)	4 (3–7)	6 (3–12)	6 (3–10)	<0.0001
**ICU length of stay (days)**				
Median (P25–P75)	3 (2–7)	7 (2–18)	6 (2–12)	0.0001

ICU, Intensive Care Unit.

Higher rates of COVID-19 hospitalization in the Brussels-Capital Region and Wallonia are observed, compared to the control group, with a significant increase across the COVID-19 waves hospitalization (*p*-trend *p* < 0.001).

In terms of co-morbidities, the proportion of obesity, hypertension and diabetes is higher among COVID-19 patients (17.3, 26.2, and 17.9%, respectively, in wave 2). Conversely, the proportion of hospitalized patients with neoplasms, pulmonary disease and smoking is lower among COVID-19 patients compared to the control group.

Regarding clinical outcomes, the percentage of ICU admission is higher for COVID-19 compared to the control group (8.1% for the control group, 20.4% for wave 1, and 17.8% for wave 2). Lengths of stay in the hospital and ICU is higher in the COVID-19 group than in the control group, with a median duration (p25-p75) of 3 (2–7) days for the control group, 7 (2–18) days for wave 1, and 6 (2–12) days for wave 2.

**Figure 1:**
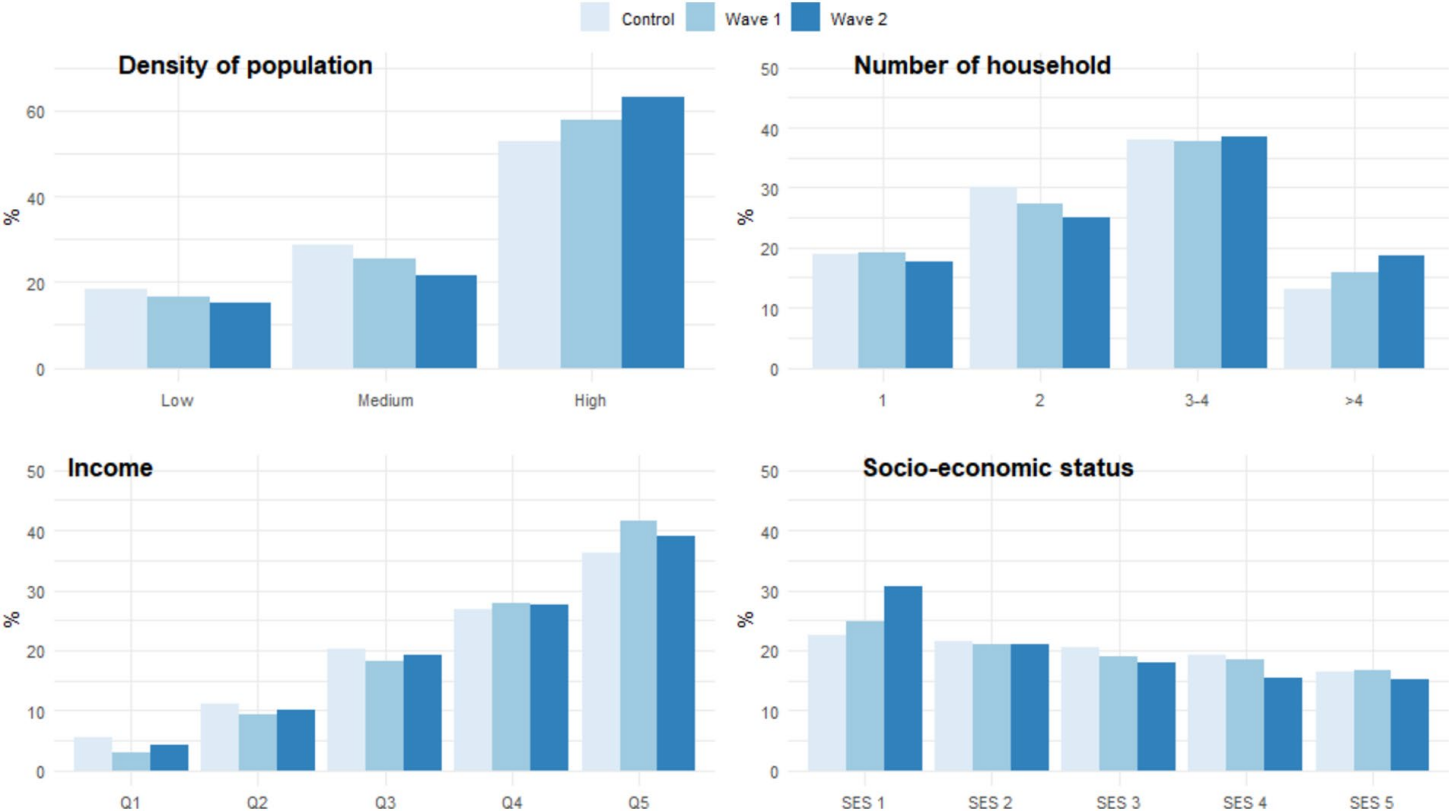
Socio-economic profiles of the control population and the population hospitalized for COVID-19 (wave 1 and 2).

A gradient was observed in each group in terms of socio-economic factors, with a significantly increased hospitalization rate with higher population densities, the lower quintile income group, and a lower SES status (p trend < 0.0001) (Figure 1). We also observed a significant increase in hospitalization across the waves and compared to the control group, for the highest density population group (p trend < 0.0001), ranging from 52.7% for the control group, 57.8% for the first wave of COVID-19, and up to 63.2% for the second wave. The same trends were observed for income and socio-economic status. The percentage of hospitalization among the most deprived group for the control group increased from 22.4 to 24.8% for wave 1, and 30.6% for wave 2 (p trend < 0.0001).

### COVID-19 hospitalized patients’ risk factors

**Figure 2:**
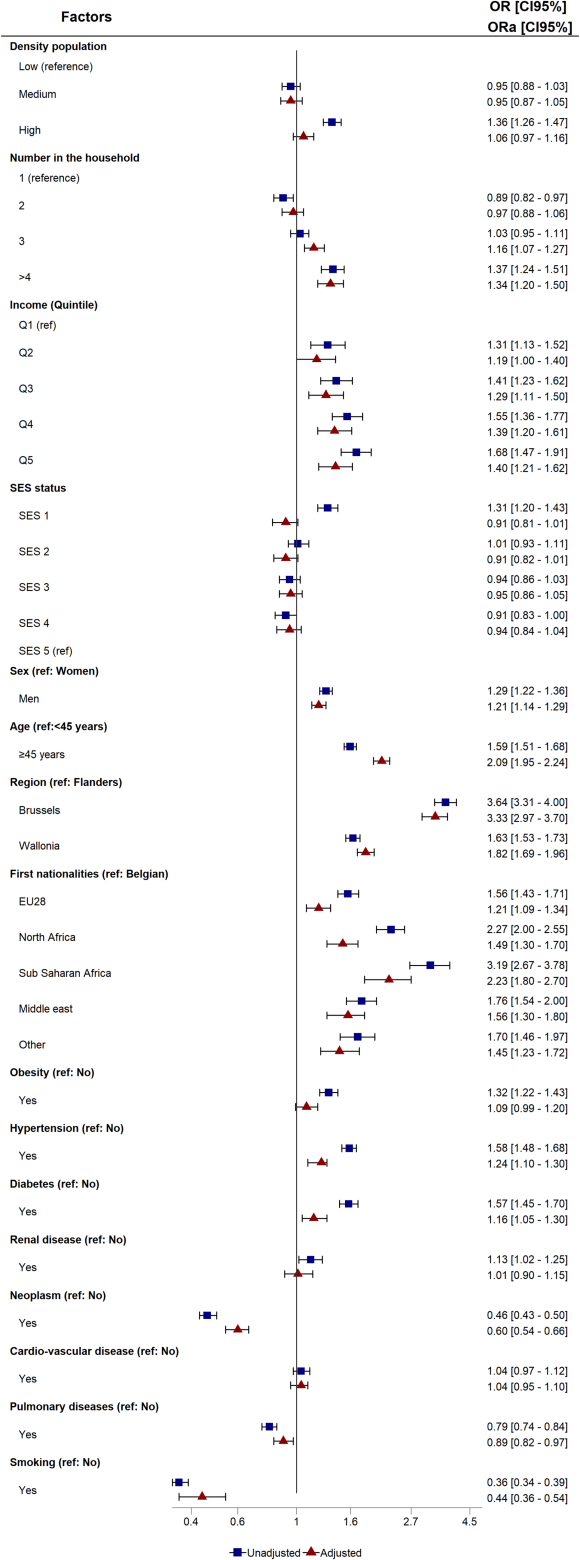
Crude and adjusted odds ratios (OR and aOR) and 95% confidence intervals of the association between COVID-19 hospitalization and risk factors (reference: control population). ICU, Intensive Care Unit; Q, Quantile; SES, Socio-Economic Status; EU28, European Union.

Figure 2 shows the crude and adjusted Odds Ratios (OR) for COVID-19 hospitalization compared to the control population, along with Supplementary Figure S2, stratified by the two waves of COVID-19.

In the univariate analyses, socio-economic factors such as high population density, more than four people per dwelling, all income quintiles, and least favorable SES status were significantly associated with COVID-19 hospitalization. An analysis by COVID-19 wave revealed a greater OR for income in wave 1 and the opposite for less favorable SES status, which was only significant in wave 2. After adjustment, population density and socio-economic status were no longer associated with COVID-19 hospitalization compared to the control population. However, for the stratified wave analysis, households with more than three individuals in wave 1 and more than three in wave 2 were found to have a higher likelihood of hospitalization. A similar pattern was observed for all the lowest-income quartiles in wave 1, and was slightly less pronounced in wave 2.

Regarding socio-demographic data, men, people over 45, and people living in Brussels and Wallonia compared with Flanders presented a significant risk factor of hospitalization for COVID-19 in both univariate and multivariate analyses. No significant differences between the two waves were observed for this socio-demographic data. All foreign nationality patients showed an increased risk of hospitalization, with a stronger association for sub-Saharan Africans in both univariate and multivariate analyses. Analysis by waves showed that nationality was only significant in wave 2, except for people from sub-Saharan Africa, who were more at risk in both waves compared to Belgians.

In terms of clinical co-morbidities, all co-morbidities were significantly associated with COVID-19 hospitalizations, with a lower risk for neoplasms, lung disease, and smoking. After adjustment, obesity and diabetes were no longer associated with COVID-19 hospitalization in the first wave, but these co-morbidities were significant in the second wave. Patients suffering from cardiovascular disease had an increased risk only in the first wave, and no association was found with kidney disease.

### COVID-19 risk factor on length of stay for hospitalized patients

**Figure 3:**
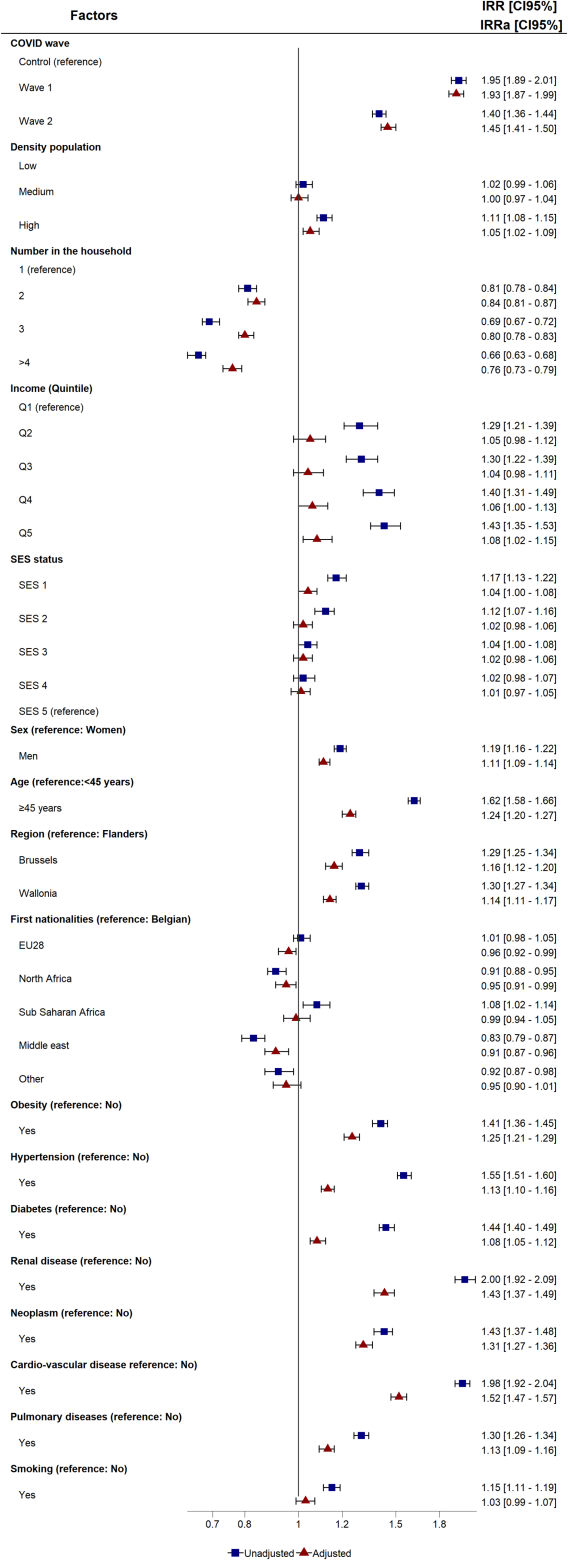
Crude and adjusted Incidence risk ratio (IRR and aIRR) and 95% confidence intervals of the association between length of stay and risk factors. ICU, Intensive Care Unit; Q, Quantile; SES, Socio-Economic Status; EU28, European Union.

Figure 3 shows the ratio of crude and adjusted relative risks for length of stay, and the Supplementary Figure S3 as stratified by population groups (COVID-19 and control).

COVID-19 patients faced a higher risk of longer hospitalization compared to the control population, with a stronger association observed in the first wave. These results remained consistent after adjustment.

Socio-economic profiles, higher population density, lower income, and less favorable SES status were significantly associated with longer lengths of stay. Conversely, higher household numbers were associated with shorter lengths of stay. As for hospitalization, the socio-economic gradient was observed for length of stay. After adjustment, the IRR of SES status and income decrease.

In both univariate and multivariate analyses, men, patients over 45 years, and residents of Brussels and Wallonia (compared with Flanders), exhibited significantly longer hospital stays. As to nationality, no significant differences were observed compared to the Belgian group except for patients from North Africa and the Middle East, with a shorter stay in both crude and adjusted IRR.

All co-morbidities were significantly associated with a longer length of stay in both univariate and multivariate analyses. However, smoking had no significance after adjustment. The analyses by population group showed a similar pattern for all risk factors by wave, and for the control population.

## Discussion

This study analyzed both clinical and social factors for COVID-19 hospitalization at an individual level, along with a pre-pandemic reference group hospitalized with non COVID-19 respiratory infections. Our study used unique linked administrative data from both hospital and social security records at an individual level and across the entire country. All patients admitted for COVID-19 in 2020 and for respiratory diseases in 2019 were included. Our results showed that the socio-economic gradient for COVID-19 followed the same pattern as the one observed in pre-pandemic respiratory diseases, with an increased risk of poorer clinical outcomes among the most disadvantaged SES groups. In addition, some risk factors were similar for hospitalization and length of stay, while others differed. Our study findings allow us to make important observations.

First, we observed an increase in the socio-economic gradient during the COVID-19 pandemic, with a temporal deterioration during the second wave in terms of hospitalization and length of stay, among the most deprived individuals. The gradient appears similar between the control population and the COVID-19 patients, but it is increased for the most vulnerable populations (low income, precarious socio-economic status, high population density area, and high number of people in the household) within the COVID-19 group. This observation confirms findings previously noted for other respiratory infections such as tuberculosis and influenza (15, 35, 36). A systematic review of health disparities during the 2009 H1N1 influenza pandemic showed that inequalities in social conditions increased exposure and risk of infection for low socio-economic status populations. COVID-19 seems to follow this pattern despite differing causes, due to the pandemic context (37). Several studies have indeed identified factors such as low education level, poverty, poor housing conditions, low family income, occupational exposure, and household overcrowding as risk factors for COVID-19 in terms of incidence, death, and confirmed diagnosis. The combination of exposure with inequalities in living and working conditions, inequality in transmission with overcrowding and dense populations, and unequal susceptibility with a higher prevalence of pre-existing health conditions in more socio-economically deprived populations have contributed to the exacerbation of social health inequalities (38). A disparity among the country’s three regions was observed, with the Brussels-Capital Region being particularly affected, at an excess mortality rate of 81.7% (123% during the first wave), more than twice that of the other two regions (21, 22). Being a multicultural city where one in three individuals lives below the poverty line, Brussels faces higher poverty risks, contributing to suboptimal healthcare access. A significant portion of the Brussels population lives in precarious socio-economic conditions, correlating with poor health (39). In Brussels, municipalities with the highest number of SARS-CoV-2 cases per population are located in the most disadvantaged and densely populated areas (19, 31). The gradient increase during the second wave is attributed to uncertainty and less frequent use of effective interventions to prevent transmission of COVID-19 (lockdown, screening, social distancing, mask use, quarantine, hand washing, etc.) in deprived populations (40–42).

Second, we observed a higher number of hospitalizations during the second wave, but with a shorter length of stay. The implemented lockdown measures varied between the waves, with stricter measures imposed during the initial wave (43). The emergence of distinct SARS-CoV-2 variants resulted in variations in transmission rates and disease severity (44). Socio-economic inequalities may have surfaced during the second wave. In Belgium, as in other European countries, the second wave exerted greater and more prolonged pressure on hospitals compared to the first wave (30, 45). Moreover, knowledge about the SARS-CoV-2 virus was more extensive, and medical teams managed COVID-19 patients more effectively during the second wave (46). Additionally, hospitals implemented different organizational strategies. During the first wave, all non-essential hospital activities were suspended. However, delays in the treatment of other diseases (e.g., cancer, chronic pathologies, etc.) were noted (47, 48), prompting hospitals to continue non-COVID-19-related activities, thereby intensifying hospital pressure during the second wave (49). As a consequence of this heightened pressure and improved understanding of the disease, lengths of stay were shorter in the second wave compared to the first (30). Furthermore, ICUs did not expand their bed capacity to the same extent, partly due to a shortage of nursing staff, which contributed to the reduction in ICU admissions during the second wave (50).

Third, we identified quite similar associated factors with hospitalization and length of stay. These factors include gender, age (individuals over 45 years old), and living in Brussels and Wallonia, compared to Flanders. Regarding gender and age, similar observations were rapidly noted for COVID-19, and explained by differences in immune response regulated by sex hormones, health behaviors, and the presence of associated co-morbidities (51, 52).

Different hypotheses could be made regarding the differential outcomes of patients depending on their region of origin. A higher prevalence of co-morbidities was noted in Brussels and Wallonia. Also, a differential adherence to non-pharmaceutical interventions was reported (53, 54). Brussels was particularly affected by the COVID-19 epidemic. The concentration of high poverty levels, dense population and a high proportion of ethnic minorities may explain higher hospitalization rates in Brussels compared to other regions (19, 31).

In our study, the association of nationality with hospitalization and length of stay is different. While all patients of foreign nationality demonstrated an increased risk of hospitalization, they exhibited a lower risk of prolonged hospital stay, except for those from Sub-Saharan Africa and the Middle East. Research on migrant health disparities during the pandemic has shown highly controversial results. European studies have described heterogeneous results; and while some found no significant differences in health outcomes due to SARS-CoV-2 infection between native and immigrant populations, others reported a higher risk.

All individuals of foreign origin had a higher hospitalization risk, as observed in Belgium and other countries (55). It is well-documented that individuals from racial/ethnic minority groups are more vulnerable to COVID-19, as has already been pointed out in previous pandemics, such as the 2009 H1N1 influenza pandemic (15, 56). This can be explained by cultural barriers, poorer health literacy, health-promoting behaviors or access to healthcare services, insecure employment and housing conditions, leisure and work-related travel, and the broader urban context, leading to greater vulnerability for certain communities to get healthcare and recover from the disease. Furthermore, certain co-morbidities are more common among individuals of other nationalities, such as diabetes in the North African population or hypertension in sub-Saharan Africans (57). However, regarding length of stay, the pattern is different: a lower risk was shown among foreign nationalities, except North Africa and the Middle East. Similar results were found with a higher risk of hospitalization for migrants but a reduced mortality odds. This could be explained as the “healthy migrant effect,” a theory that has been thoroughly discussed in literature (58).

Other co-morbidities, such as obesity, hypertension or diabetes, have known risk factors for hospitalization and increased length of stay (59, 60). Neoplasms and pulmonary pathologies, on the other hand, were inversely protective for hospitalization, but associated with a longer length of stay, possibly explained by greater caution among these individuals, and adherence to non-pharmacological ways to prevent infection (61). Smoking is known to be a risk factor for hospitalization and poor prognosis for COVID-19 (62, 63). Our results do not entirely align with these observations, showing a lower risk of hospitalization and association with length of stay, which could be explained by the study design and its specificities regarding the control group, or an unidentified confounding factor (64).

Based on our findings, several recommendations may be made. First, it is crucial to strengthen public health interventions targeting vulnerable populations. Second, improving access to social health data is essential to better understand the needs of disadvantaged communities. Additionally, it is important to emphasize the significance of systematically collecting social data alongside clinical data, as this will enable a more comprehensive analysis of population health. Furthermore, investing in research to better understand the mechanisms of social health inequalities is necessary. A thorough understanding of these mechanisms is vital to inform policymakers and guide public policies toward more effective solutions. Finally, considering the increased length of stay for COVID-19 patients, particularly during the second wave and in the Brussels region, as well as among the most deprived patients, a geographical distribution of patients based on their socio-economic factors could lead to a better distribution of infected patients. This approach would contribute to a more balanced distribution of resources and more effective management of those affected by the disease.

## Limits and strengths

This study presents limitations. First, the encoding of diagnostic codes within hospital data may introduce inaccuracies or misclassifications, potentially impacting the reliability of our findings. Second, it’s important to note that medical administrative data is primarily collected for hospital financing purposes rather than epidemiological studies, which could introduce biases or limitations in the data’s suitability for our research objectives. Additionally, due to data protection regulations such as General Data Protection Regulation (GDPR) certain categories may have been clustered, potentially limiting the granularity of our analysis and introducing challenges in assessing specific socio-economic factors. Moreover, the observational nature of our study design precludes establishing causality, and there may be unmeasured confounding factors influencing the observed associations. While efforts were made to adjust for potential confounders, residual confounding may still be present. Lastly, another significant limitation of our study is that the linkage based on the pseudonymised national registry number excludes all individuals who are not officially residents of Belgium. This population, including undocumented migrants and the homeless, remains systematically invisible in administrative data. Their exclusion is particularly concerning as they are highly vulnerable, living in precarious conditions with limited access to healthcare and health insurance. Previous exploratory studies conducted in public hospitals in the most deprived and multiethnic areas of Brussels have shown a higher risk of COVID-19 hospitalization and ICU admission among individuals without health insurance (19, 31).

Despite these limitations, our study has several strengths. First, our analysis is conducted at an individual level, allowing for a detailed examination of socio-economic disparities and their interaction with COVID-19 outcomes. Second, our study includes all hospitalized patients, providing a population-based perspective that enhances representativeness and generalisability of our findings. Moreover, our approach integrates both social and clinical factors, allowing for a comprehensive understanding of the complex interplay between socio-economic status and COVID-19 outcomes. These strengths contribute to the robustness and relevance of our study in informing public health strategies that were aimed at addressing health disparities during the COVID-19 pandemic.

## Conclusion

This study provides robust evidence supporting the presence of a social health gradient that mirrors that of pre-pandemic respiratory diseases, intensified in the second waved and among the most deprived groups. Our findings reveal that various factors of socio-economic status were consistently associated with both hospitalization rates and lengths of hospital stays among COVID-19 patients, in comparison to a control group. In light of these results, which highlight evidence that non-medical factors are critical determinants of health outcomes, we recommend integrating socio-economic disparities into public health strategies aimed at addressing them during pandemics. Additionally, we suggest implementing a policy for accessing on both social and health data to enable real-time monitoring of the effects of pandemics on population health.

## Data Availability

The original contributions presented in the study are included in the article/Supplementary material, further inquiries can be directed to the corresponding author.
