# Supplementary material for: Social health gradient and risk factors among patients hospitalized for COVID-19 and pre-pandemic respiratory infections. A linked national individual case-control study in Belgium
Source: Front Public Health. 2024 Oct 28;12:1426898. doi: 10.3389/fpubh.2024.1426898 (PMC11551126; doi:10.3389/fpubh.2024.1426898)
Supplement: Supplementary file 1 [file Data_Sheet_1.docx]

Supplementary Material

**Supplementary data, Figure 1:** Flow Chart


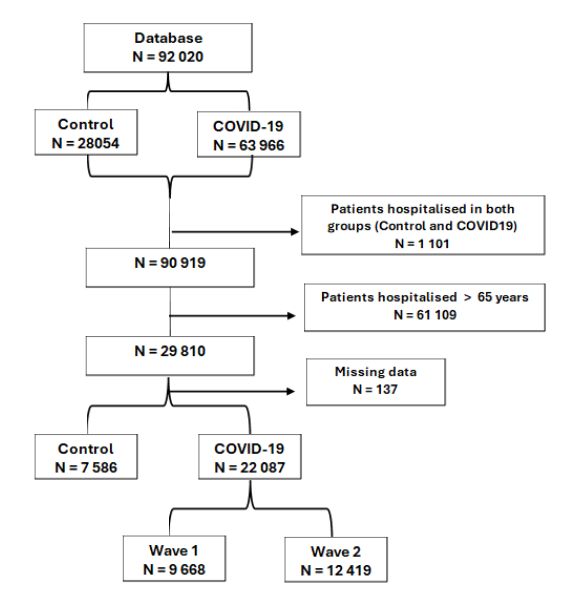


**Supplementary data, Figure 2:** Crude and adjusted Relative Risk Ratios (RRR and aRRR) and 95% confidence intervals between COVID-19 hospitalisation (wave 1 and wave 2) and risk factors (reference: control population)


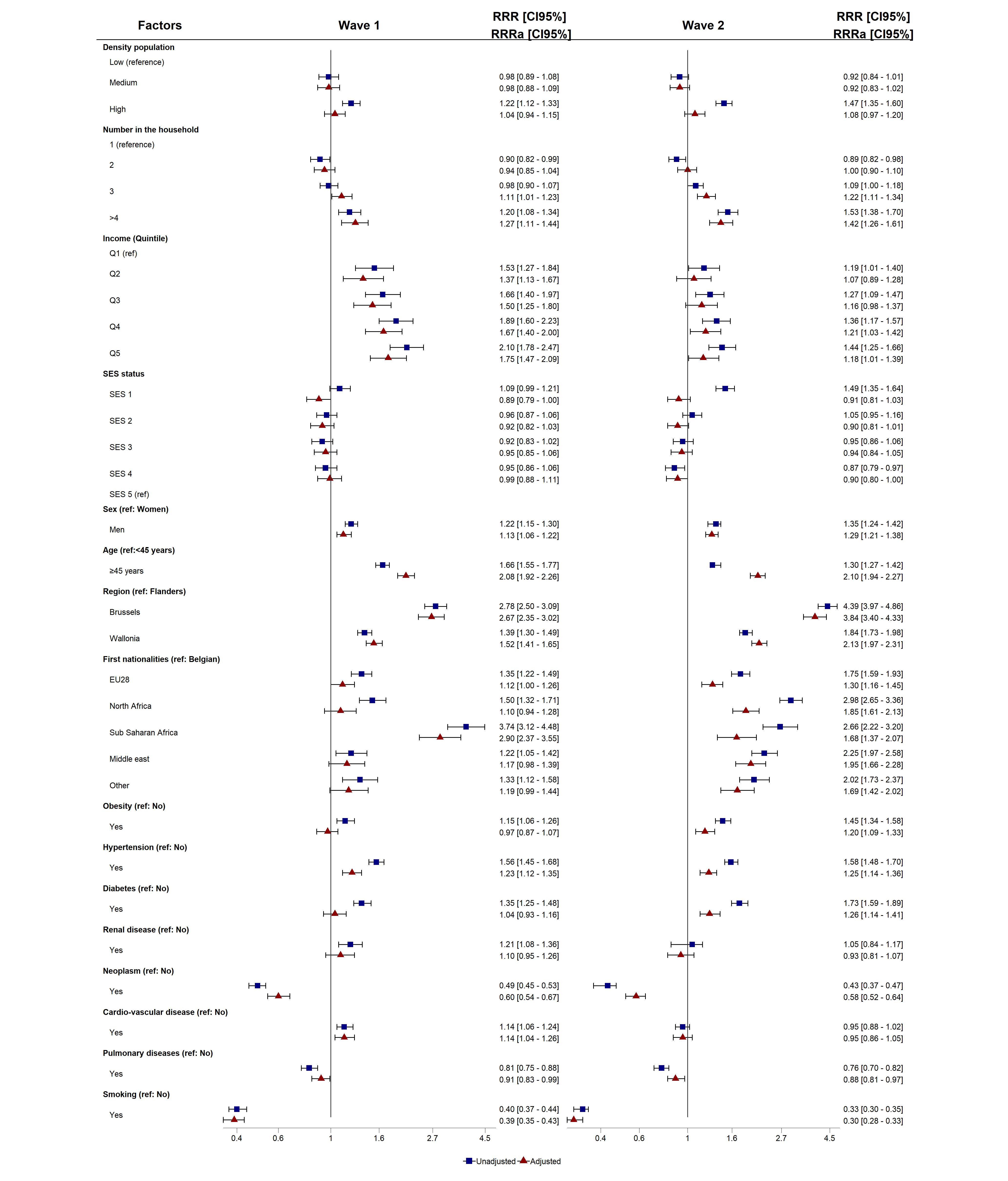


Legend: ICU: Intensive Care Unit; Q: Quantile; SES: Socio-Economic Status; EU28: European Union

**Supplementary data, Figure 3:** Crude and adjusted Incidence risk ratio (IRR and aIRR) and 95% confidence intervals of the association between length of stay and risk factors stratified by group population (COVID-19 and control)


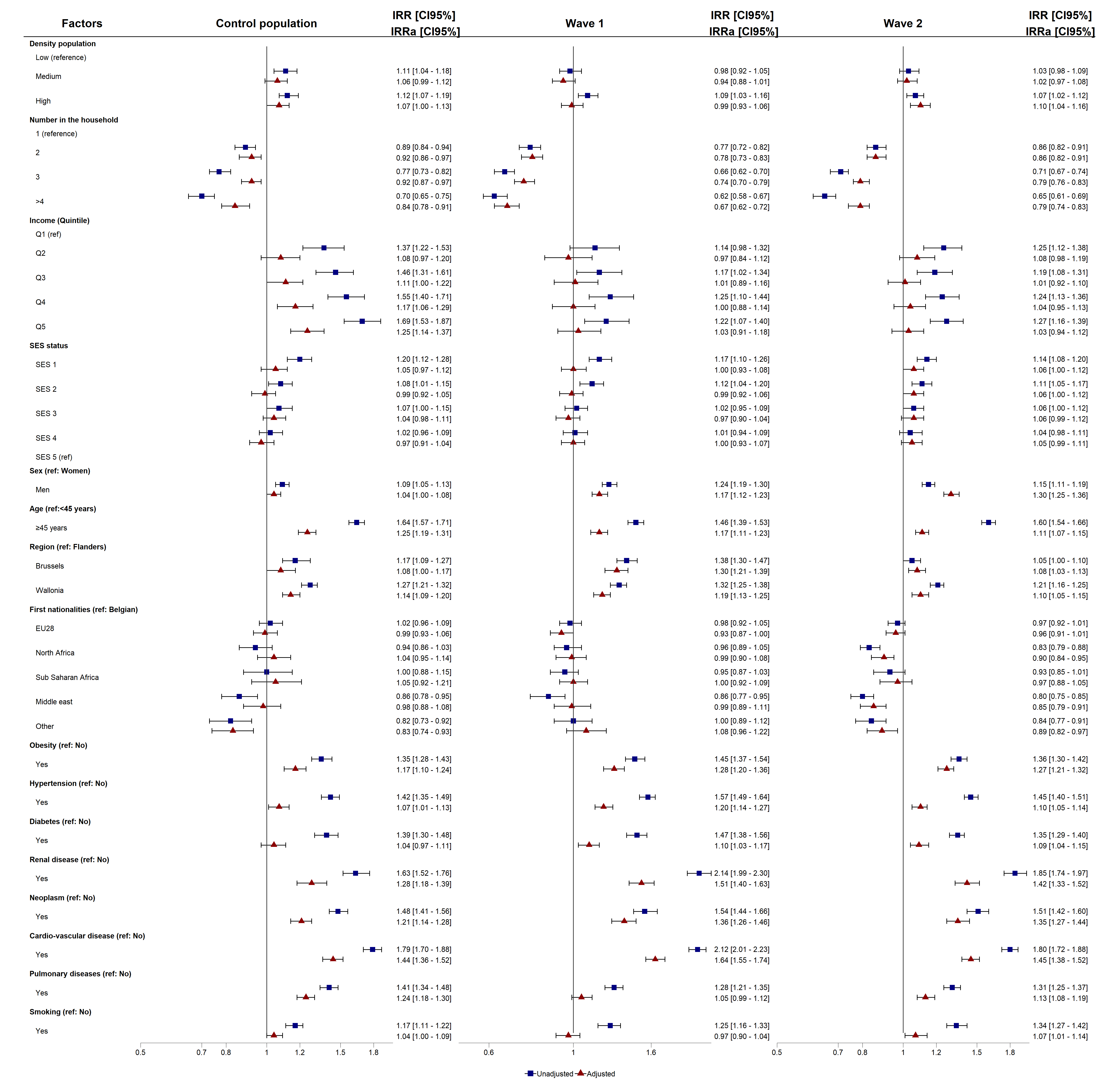
Legend: ICU: Intensive Care Unit; Q: Quantile; SES: Socio-Economic Status; EU28: European Union
